# The Antibiofilm Effect of a Medical Device Containing TIAB on Microorganisms Associated with Surgical Site Infection

**DOI:** 10.3390/molecules24122280

**Published:** 2019-06-19

**Authors:** Valentina Puca, Tonino Traini, Simone Guarnieri, Simone Carradori, Francesca Sisto, Nicola Macchione, Raffaella Muraro, Gabriella Mincione, Rossella Grande

**Affiliations:** 1 Department of Medicine and Aging Science, “G. d′Annunzio” of Chieti-Pescara, Via dei Vestini 31, 66100 Chieti, Italy; valentina.puca@unich.it; 2 Center of Excellence on Aging and Translational Medicine (CeSI-MeT), Via Luigi Polacchi 11, 66100 Chieti, Italy; simone.guarnieri@unich.it; 3 Department of Medical, Oral, and Biotechnological Sciences, “G. d’Annunzio” University of Chieti-Pescara, Via dei Vestini 31, 66100 Chieti, Italy; tonino.traini@unich.it (T.T.); raffaella.muraro@unich.it (R.M.); gabriella.mincione@unich.it (G.M.); 4 Department of Neuroscience Imaging and Clinical Sciences, “G. d′Annunzio” University Chieti-Pescara, Via Luigi Polacchi 11, 66100 Chieti, Italy; 5 Department of Pharmacy, “G. d’Annunzio” University of Chieti-Pescara, Via dei Vestini 31, 66100 Chieti, Italy; simone.carradori@unich.it; 6 Department of Biomedical, Surgical and Dental Sciences, University of Milan, Via della Commenda 10, 20122 Milan, Italy; francesca.sisto@unimi.it; 7 Department of Urology, University of Milan, ASST Santi Paolo e Carlo, Via Antonio di Rudinì 8, 20142 Milan, Italy; nicola.macchione@asst-santipaolocarlo.it

**Keywords:** *Staphylococcus aureus*, *Enterococcus faecalis*, antimicrobial activity, antibiofilm activity, surgical site infection, TIAB

## Abstract

Surgical site infections (SSIs) represent the most common nosocomial infections, and surgical sutures are optimal surfaces for bacterial adhesion and biofilm formation. *Staphylococcus* spp., *Enterococcus* spp., and *Escherichia coli* are the most commonly isolated microorganisms. The aim of this research was to evaluate the antibiofilm activity of a medical device (MD) containing TIAB, which is a silver-nanotech patented product. The antibacterial effect was evaluated against *Staphylococcus aureus* ATCC 29213, *Enterococcus faecalis* ATCC 29212, and *E. coli* ATCC 25922 by assessing the minimum inhibitory concentration (MIC) by the Alamar Blue^®^ (AB) assay. The antibiofilm effect was determined by evaluation of the minimum biofilm inhibitory concentration (MBIC) and colony-forming unit (CFU) count. Subsequently, the MD was applied on sutures exposed to the bacterial species. The antimicrobial and antibiofilm effects were evaluated by the agar diffusion test method, confocal laser scanning microscopy (CLSM), and scanning electron microscopy (SEM). The MIC was determined for *S. aureus* and *E. faecalis* at 2 mg/mL, while the MBIC was 1.5 mg/mL for *S. aureus* and 1 mg/mL for *E. faecalis*. The formation of an inhibition zone around three different treated sutures confirmed the antimicrobial activity, while the SEM and CLSM analysis performed on the MD-treated sutures underlined the presence of a few adhesive cells, which were for the most part dead. The MD showed antimicrobial and antibiofilm activities versus *S. aureus* and *E. faecalis*, but a lower efficacy against *E. coli*. Surgical sutures coated with the MD have the potential to reduce SSIs as well as the risk of biofilm formation post-surgery.

## 1. Introduction

Surgical site infections (SSIs) are classified as ”infections occurring within 30 days after a surgical operation (or within one year if an implant is left in place after the procedure) and affecting either the incision or deep tissue at the operation site” [1,2]. SSIs represent the most common nosocomial infections and account for 14–16% of all healthcare-associated infections among hospitalized patients and 38% among surgical patients [2,3]. SSIs are the cause of morbidity and in some cases mortality [4], and increase healthcare costs, since they imply a prolonged hospitalization, additional diagnostic tests, massive antibiotics use, and sometimes invasive surgery. Surgical sutures represent a suitable substrate for microbial adhesion and, therefore, for biofilm formation. A significant rate of hospital-acquired infections is associated with biofilms developed by pathogens and/or opportunistic pathogens. A biofilm is a microbial community protected by a self-produced complex matrix of extracellular polymeric substances (EPS) adherent to each other and/or to a surface that protects the microorganisms from the attack by the antimicrobials and the host immune system [5,6].

As reported by Koo et al., the biofilm was initially described as the “arcane behavior of bacterial populations”, but it is currently described as a “principle virulence factor in many localized chronic infections”. The infections associated with biofilm usually develop after a long period of hospitalization, and the microbial community of the biofilm may develop tolerance to the antibiotics using different strategies such as the entry into a dormancy or viable but non-culturable (VBNC) status or the development of molecular pathways that guarantee their persistence and survival [7]. Therefore, the biofilm EPS matrix plays an important role in conferring antimicrobial tolerance to biofilms [6,7]. The bacteria more commonly isolated by SSIs are *Staphylococcus* spp., *Enterococcus* spp., and *E. coli*; however, the microorganisms that are isolated could depend on the type of surgery [2,8]. Many approaches have been developed for the in vitro treatment of the biofilms.

Metals and metal oxide nanomaterials have been widely studied for their applications in the clinical field because of their innovative size and properties. Titanium dioxide (TiO_2_) nanotubes have been used such as for biosensors, drug delivery systems, and cancer therapy [9]. The antimicrobial activity of metals such as silver (Ag), copper, gold, titanium, and zinc, which are characterized by different properties and spectra of action, have been known for centuries [9,10]. Aydin et al. demonstrated that Ag-doped TiO_2_ nanotubes could be used as an effective material that is free of antimicrobial resistance [9]. Other researchers showed that TiO_2_ microspheres combined with Ag particles possess antimicrobial properties, particularly versus *S. aureus* and *E. coli*, suggesting an application in dentistry, orthopedics, and other field of medicine [11,12]. However, TiO_2_ as well as ZnO nanomaterials are the most studied for their activity not only toward bacterial infections, but also toward viral, fungal, and protozoal infections [13]. The antimicrobial activity is performed through the induction of the production of reactive oxygen species (ROS), which damage the cellular membrane and cause the peroxidation of proteins and lipids. Recently, Peiris et al. showed that the antimicrobial activity of TiO_2_ nanoparticles biosynthesized by using Backer’s yeast was greater versus Gram-positive bacteria and *Candida albicans* than versus Gram-negative ones [14]. Noble metals such as Ag are often used to modify oxide-based semiconductors for improving the antimicrobial activity [15].

Furthermore, silver nanoparticles are widely studied for their application in wound healing because of their physicochemical and biological properties. Silver nanoparticles are non-cytotoxic and safe, and can be used for the preventive control of the growth of microorganisms in the site of infection [16,17]. Nanoparticles are considered an alternative strategy to the use of the common antibiotics. It was demonstrated that nanoparticles work discharging toxic metal ions or by inducing the production of reactive oxygen species (ROS) [17]. The negatively-charged groups on the bacterial surfaces attracted positively charged nanoparticles, inducing the formation of channels on the bacterial cell wall [18,19]. Nanoparticles are capable of penetrating the bacterial cell wall and affecting the metabolic pathways and proton efflux pumps, inducing a modification of the pH and membrane surface charges [20]. The efficacy of nanoparticles used alone or functionalized with antibiotics or natural compounds have been studied for application in wound healing [19]. In particular, silver nanoparticles have been studied for wound dressing in pressure ulcers or coat polyester–nylon wound dressing for reducing the risk of *Pseudomonas aeruginosa* and *S. aureus* infections [21,22].

The main aim of the present investigation was to establish the antibiofilm activity of a medical device (MD) containing TIAB, which consists of microcrystalline titanium dioxide (TiO_2_) nanoparticles (100 to 500 nm) covalently linked with monovalent silver ions (Ag^+^), commercially available under the trademark Peonil^®^. The antibiofilm activity was evaluated on microorganisms, biofilm-producers, associated with SSIs in the male urogenital tract. The medical device used is characterized by different components with complementary pharmacological activities such as TIAB, which is the silver preparation, *Aloe vera* extract, and hyaluronic acid. The other two major components were shown to display anti-inflammatory and healing effects on damaged epithelium, forming a protective barrier against external noxae. This complex mixture helps treating/slowing infections caused by bacteria, fungi, and viruses, including the genital herpes virus (HSV-2). It is also involved in tissue regeneration and wound healing after surgery. To the best of our knowledge, this medical device has been never tested for its antimicrobial/antibiofilm activity or its efficacy after treatment on three commercially available braided surgical sutures exposed to different microorganisms despite its use in the clinical treatment/prevention of SSIs. The sutures were selected on the basis of their common use after surgery and their differences in terms of material, absorption, and diameter.

## 2. Results and Discussion

**Figure 1:**
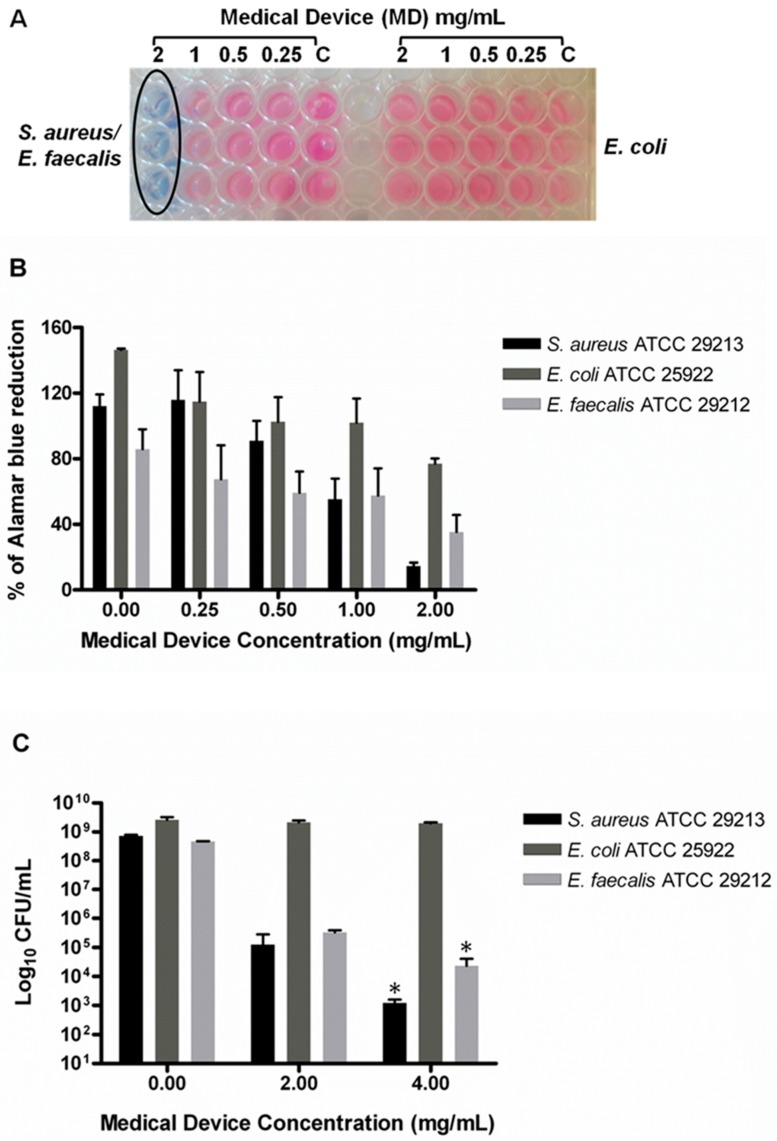
Evaluation of minimum inhibitory concentration (MIC) and minimum bactericidal concentration (MBC) of the medical device (MD) versus *S. aureus*, *E. coli*, and *E. faecalis.* The MIC was determined by Alamar Blue^®^ (AB) assay, which is based on the chemical reduction of resazurin (a non-toxic, cell-permeable, non-fluorescent, blue compound) in resorufin, which is a highly fluorescent red/purple compound. Such reduction is performed by viable cells; therefore, continued cell growth maintains a reduced environment, while the inhibition of growth or cell death induces an oxidized environment. (**A**) Representative image of colorimetric MIC determination using AB assay. The black circle indicates the MIC at 2 mg/mL which is the same for both *S. aureus* and *E. faecalis*. (**B**) The plot shows the percentage reduction of AB recorded for the three bacterial species at different MD concentrations compared to the corresponding untreated samples (0.00) performed as described in the Materials and Methods section. The percentage of AB reduction was evaluated by using absorbance at 570 nm and 600 nm. (**C**) The MBC was determined by CFU counts and corresponds to 4 mg/mL for *S. aureus* and *E. faecalis*. Data are the mean of three replicates of three independent experiments; * *p* < 0.05 vs. the controls (0.00). C: controls or untreated samples.

The antimicrobial and antibiofilm effects of the MD were evaluated against three reference strains: *S. aureus* ATCC 29213, *E. faecalis* 29212, and *E. coli* 25922 (Table 1). The minimum inhibitory concentration (MICs) were determined by using the broth micro-dilution method, according to the Clinical and Laboratory Standards Institute (CLSI) guidelines. The results obtained were confirmed by the AB reduction assay, which represents a cell health indicator. The kit contains resazurin, which is a non-toxic, cell-permeable, blue, non-fluorescent molecule that is reduced to resorufin, which is red in color, and is a fluorescent compound when the cells are alive (Figure 1A,B). The MD displayed an MIC corresponding to 2 mg/mL versus *S. aureus* and *E. faecalis* (Table 1 and Figure 1A,B); on the contrary, no antimicrobial effect was detected against *E. coli*, which showed MIC >8 mg/mL.

With regard to MBC, the MD was shown to be effective at 4 mg/mL versus *S. aureus* and *E. faecalis* (Table 1); this was different from *E. coli*, which showed MBC > 8 mg/mL (Table 1; Figure 1C). The MD showed antimicrobial and antibiofilm activities versus *S. aureus* and *E. faecalis*, but a lower efficacy against *E. coli*. The antimicrobial activity is definitely due to the silver-based component (TIAB), because the antibacterial activity exerted by silver ions as well as by TiO_2_ nanoparticles has long been known [13,23]. TIAB can be used as an effective topic therapeutic agent in the treatment of chronic periodontitis along with supportive periodontal therapy and after ocular surgery. Lauritano et al. demonstrated the antimicrobial effect of TIAB against red complex organisms; in particular, a significant reduction of *Treponema denticola* and *Porphyromonas gingivalis* was observed [24]. The effect of TIAB has been the topic of medical studies, especially in the dermatological field [25]. Silver ions are capable of inducing bacterial death by different pathways: (i) bacterial proteins inactivation, (ii) the inhibition of DNA replication, (iii) the modulation of gene expression, (iv) blockade of the electron transport chain with the consequent reduction of ATP, and ultimately, interference with signaling pathways, which are essential for bacterial survival [23]. Since the MD has shown efficacy exclusively toward *S. aureus* and *E. faecalis*, the minimal biofilm inhibitory concentration (MBIC) was evaluated versus these two bacterial species. As regards growth inhibition, it is not unexpected to register limited activity against Gram-negative species due to both the presence of an outer membrane that is difficult to penetrate and overexpressed efflux pumps.

**Figure 2:**
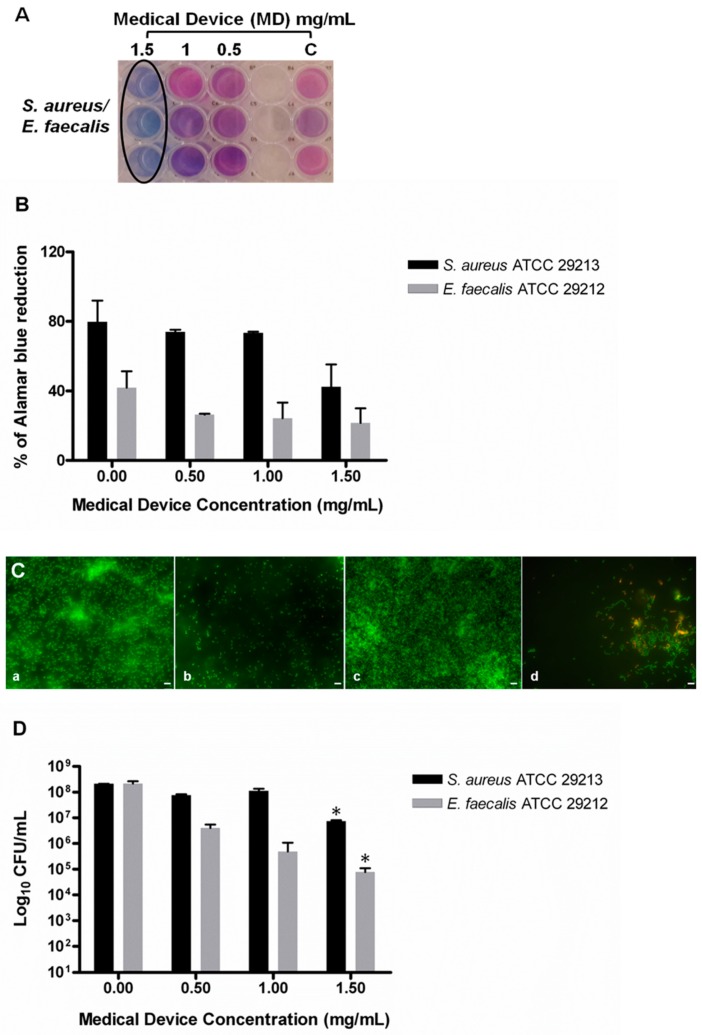
Evaluation of minimum biofilm inhibitory concentration (MBIC) of the medical device (MD) versus *S. aureus* and *E. faecalis* biofilm determined by AB assay, live/dead staining, and fluorescent microscopy analysis and colony-forming unit (CFU) counts. (**A**) Representative image of colorimetric MIC determination using AB assay. The black circle indicates the MBIC at 1.5 mg/mL, which is the same for both *S. aureus* and *E. faecalis*. (**B**) The plot shows the percentage reduction of AB recorded for *S. aureus* and *E. faecalis* at different MD concentrations compared to the corresponding untreated samples (0.00), as previously described. The percentage of AB reduction was evaluated by using absorbance at 570 nm and 600 nm. (**C**) Representative images of live/dead staining of *S. aureus and E. faecalis* biofilm developed with and without the addition of MD at the inoculum. *S. aureus* biofilm after 24 h of incubation: (a) *S. aureus* treated with 1.5 mg/mL of MD at the inoculum, (b) *E. faecalis* biofilm after 24 h of incubation, (c) *E. faecalis* treated with 1 mg/mL of MD, (d) the green fluorescence indicates live cells; the samples treated with the MD did not develop a biofilm, as shown in images (b and d). bar: 5 μm. (**D**) Evaluation of the CFU number in the MD-treated and untreated sample. A significant reduction was detected at 1.50 mg/mL in both the microorganisms. Data are the mean of three replicates of three independent experiments; * *p* < 0.05 vs. the controls (0.00). C: controls or untreated samples.

The MD also showed the capability of inhibiting the development of a mature biofilm by *S. aureus* and *E. faecalis*. In particular, the MBIC was determined by using AB assay, CFU counting, and live/dead staining, followed by fluorescent microscopy analysis. The MD displayed MBIC at 1.5 mg/mL toward *S. aureus* and 1 mg/mL toward *E. faecalis* (Table 1 and Figure 2A,B). The microscopy analysis showed the development of a mature biofilm for both strains after 24 h of incubation (Figure 2C, a and c). On the contrary, the samples treated with the MD showed no biofilm formation with few live cells adhered onto the plate surface (Figure 2C). The inhibition of the biofilm development in the treated samples was confirmed by a statistical significant reduction of CFU compared to the controls (Figure 2D, b and d). The capability of MD to inhibit biofilm formation is probably due by interfering with quorum sensing. It has been demonstrated that silver ions are capable of penetrating through the biofilm matrix, where they are less susceptible to modifications compared to common drugs [26]. The Ag^+^–TiO_2_ nanoparticles are able to interfere with the microbial quorum sensing affecting the biofilm development [23]. The surgical sutures represent an optimal substrate for the bacterial cells adhesion, and thus biofilm development. Therefore, the capability of MD to inhibit *S. aureus* and *E. faecalis* biofilm formation was evaluated on three sutures having different characteristics. The sutures were treated and non-treated with the MD as indicated in the Material and Methods section.

**Figure 3:**
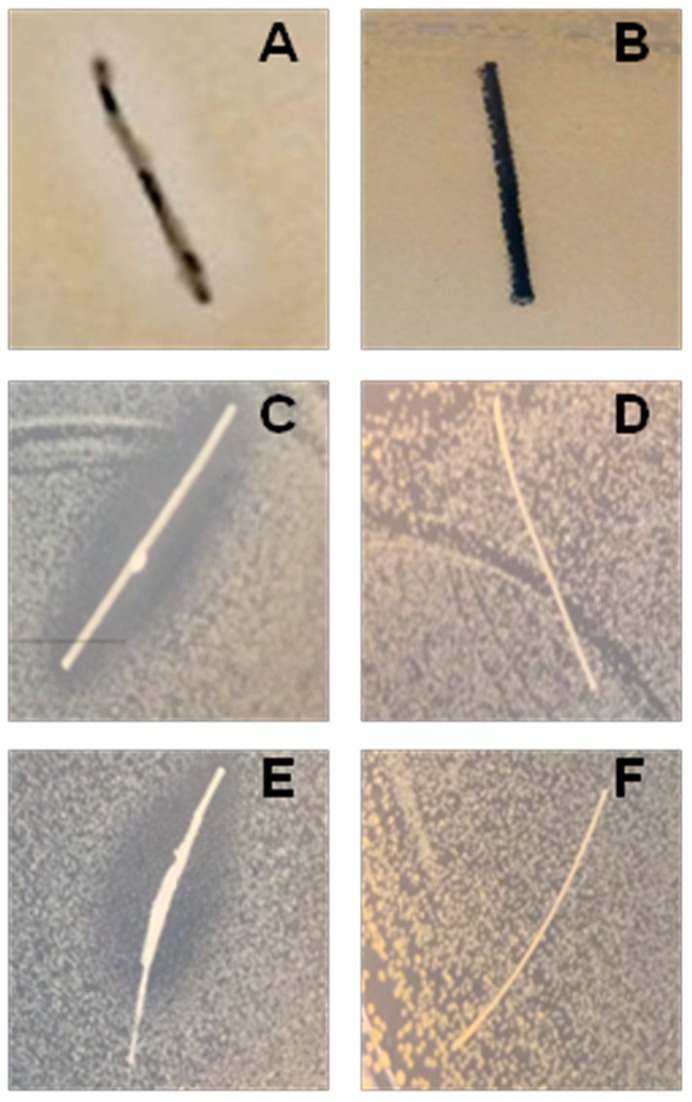
Representative images of the inhibition zones on Mueller-Hinton agar (MHA) plates of the three different surgical sutures treated (**A**, **C**, **E**) and untreated (**B**, **D**, **F**) with the medical device (MD). The efficacy of the MD versus *S. aureus* is demonstrated by the presence of an inhibition zone around the treated sutures. Similar results have been obtained versus *E. faecalis.*

**Figure 4:**
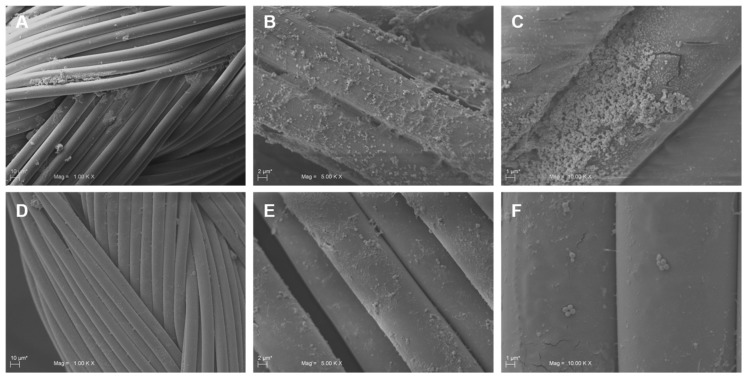
Representative SEM images of sutures untreated and treated with the medical device (MD). Panels **A**, **B**, and **C** represent different magnifications of untreated sutures with *S. aureus* cells adhered on the surface of the threads. *S. aureus* aggregates forming a well-developed biofilm were visible on panels **B** and **C**. On the contrary, few scattered cells were attached on the suture surface of MD-treated samples (**D**, **E**, **F**).

**Figure 5:**
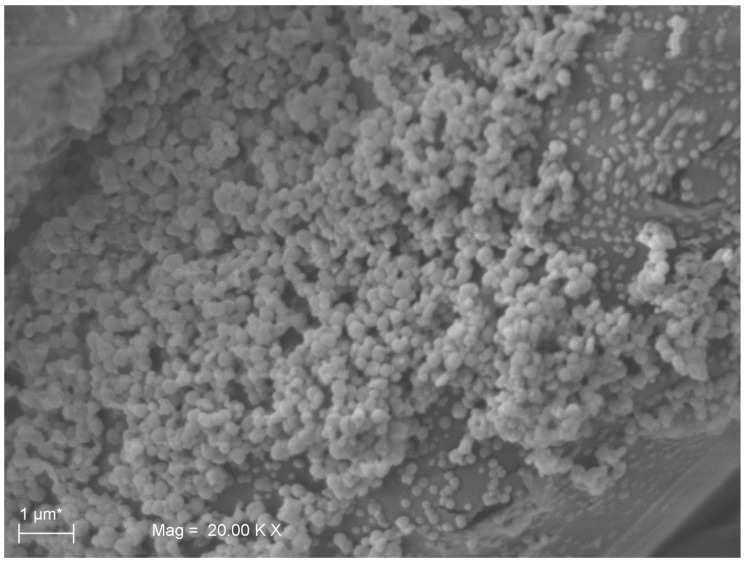
Representative SEM image a well-structured biofilm developed by *S. aureus* on suture thread. The image shows the *S. aureus* capability of forming an abundant biofilm on sutures without medical device (MD) treatment.

**Figure 6:**
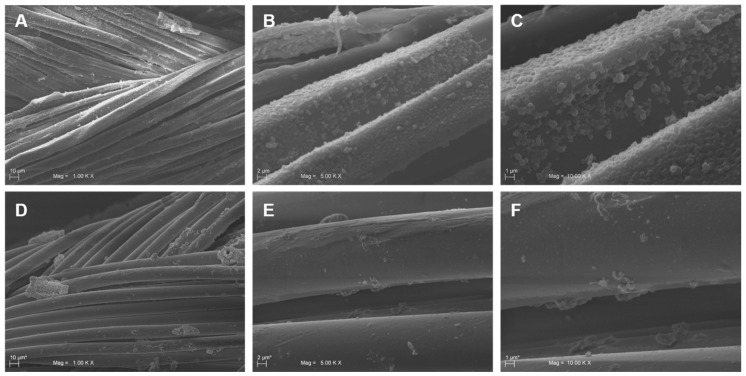
Representative SEM images of sutures untreated and treated with the medical device (MD). Panels **A**, **B**, and **C** represent different magnifications of untreated sutures with *E. faecalis* cells adhered on the surface of the threads. *E. faecalis* aggregates forming a biofilm were visible on panels **B** and **C**. On the contrary, few cells were attached on the suture surface of MD-treated samples (**D**, **E**, **F**).

The efficacy of the MD was demonstrated by the agar diffusion test method, scanning electron microscopy (SEM) and confocal laser scanning microscopy (CLSM). The formation of an inhibition zone around the treated multifilament sutures confirmed the MD antimicrobial activity; in fact, TIAB is able to diffuse in the agar inhibiting the *S. aureus* growth (Figure 3A,C and E), while the SEM and CLSM analysis underlined the presence of few adhered cells on the MD-treated sutures with respect to the controls, which showed a well-structured biofilm characterized by numerous cell aggregates (Figure 4 and Figure 5). The same results were obtained against *E. faecalis* (Figure 6).

**Figure 7:**
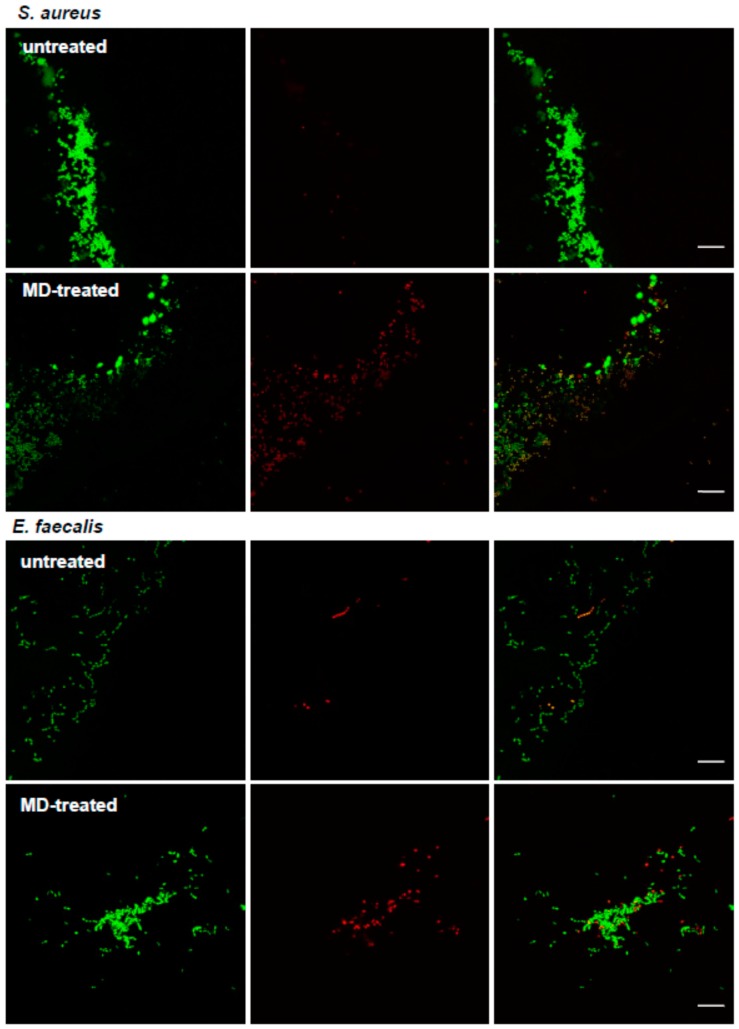
Representative images obtained by confocal laser scanning microscopy (CLSM) of *S. aureus* and *E. faecalis* grown on sutures untreated and MD-treated, stained with BacLight kit (bar: 10 µm). Green fluorescence indicates live cells, and red fluorescence indicate dead cells. In the untreated samples, aggregates of live cells organized in a biofilm were visible. On the contrary, in the MD-treated sutures, an increased number of dead cells was present.

The data obtained demonstrated that bacteria adhere to these braided multifilament sutures, although they prefer suture threads of silk composed of an organic protein called fibroin with respect to synthetic ones (data not shown). The Alamar Blue reduction as well as the CFU counting and live/dead assay displayed the efficacy of the MD in protecting the suture threads from microbial colonization and biofilm formation (for *S. aureus* and *E. faecalis*). The SEM and CLSM analysis confirmed the presence of a few bacterial cells, most of which were dead (indicated by the red fluorescence), and adhered on MD-coated sutures compared to the non-treated controls, which showed the presence of a well-structured biofilm characterized by live cells (green fluorescence) (Figure 7). However, the data obtained can be underestimated, since the treated suture threads have been immersed in liquid medium; we cannot exclude a reduction in the MD effect due to dissolution of the cream in the culture medium. In the same way, we cannot exclude the possible adhesion of bacterial cells in the stitches of the suture thread not covered by cream. The interesting aspect is that TIAB explicates its antibacterial and antibiofilm activity even when complexed with other components in the form of cream, providing that this combination does not inhibit its efficacy. In addition, no differences in the biofilm formation were registered on the three different braided multifilament sutures, thus confirming the broad MD versatility after surgery, as demonstrated for triclosan-coated sutures [27].

SSIs are responsible for complications after surgical operations despite antibiotic prophylaxis [28,29]. SSIs represent 2–5%, but can reach 25%, as in the case of colorectal surgery [30]. Therefore, the use of antimicrobial-coated sutures such as the ones coated with triclosan [29,31] might represent a possible approach. Several studies demonstrated the in vitro antimicrobial effect of triclosan [32,33]. However, some studies demonstrated the development of bacterial resistance versus triclosan, hypothesizing the possible selection of resistant bacterial strains [34,35]. In addition, Obermeier et al. demonstrated the antimicrobial efficacy of chlorhexidine and octenidine applied on surgical sutures, particularly versus *S. aureus* [36]. The antimicrobial and antibiofilm activities of a commercially available MD (Peonil^®^), in the form of cream, when applied to the suture threads, represent a valid strategy to reduce the risk of infection, particularly by biofilm-producer microorganisms. Furthermore, the application of a cream formulation on surgical sutures could avoid the use of coated sutures, which are more expensive and can induce side effects to the patients. The therapy could be monitored and tailored according to the patient allowing the easy removal of the cream in case of topical toxicity or allergy. We focalized the attention on bacterial species associated with infections of male urogenital tract, since the present work represents the basis for the start of a clinical trial to be implemented in male patients undergoing surgical operations in the urogenital tract. However, the efficacy of the MD should be demonstrated also against microorganisms associated with infections in other surgical sites.

## 3. Materials and Methods

### 3.1. Bacterial Strains and Culture Conditions

The antimicrobial activity of the medical device containing TIAB was evaluated on three reference strains: *S. aureus* ATCC 29213, *E. faecalis* ATCC 29213, and *E. coli* ATCC 25922. The strains were cultured on Mueller-Hinton agar (MHA, Oxoid Ltd., Hampshire, UK), Mannitol salt agar (MSA, Oxoid Ltd., Hampshire, UK), and MacConkey agar (MCKA, Oxoid Ltd., Hampshire, UK), respectively.

The reference strains were conserved at −80 °C before being melted at room temperature and plated on tryptic soy agar (TSA; Oxoid Ltd., Hampshire, UK). Then, bacteria were grown in tryptic soy broth (TSB; Oxoid Ltd., Hampshire, UK) for 16 h at 37 °C under shaking conditions at 125 rpm (Innova 4300, New Brunswick Scientific, Edison, NJ, USA).

### 3.2. Determination of Minimum Inhibitory Concentration (MIC) and Minimum Bactericidal Concentration (MBC)

The MIC and MBC were determined in Mueller-Hinton broth (MHB, Oxoid Ltd., Hampshire, UK) by using the broth microdilution method in 96-well polystyrene microtiter plates (Eppendorf, Hamburg, Germany) according to the Clinical and Laboratory Standards Institute (CLSI) [37]. The overnight broth cultures were resuspended in Mueller-Hinton broth to an optical density (OD_550_) of 0.8 at 550 nm corresponding to 1.0 × 10^8^ colony-forming units (CFU)/mL. The broth cultures were diluted 1:100 in MHB and then 1:10 to reach a final concentration of 1.0 × 10^5^ CFU/mL according to the CLSI guidelines. The MD Peonil^®^ (generously provided by Idi Pharma Srl, Catania, Italy) was diluted in MHB to obtain the range of concentrations of 0.25 to 8 mg/mL. Controls included: (i) broth cultures in MHB without the addition of MD; (ii) MHB with MD at different concentrations, and (iii) just MHB. The plates were put into an incubator at 37 °C for 24 h. Three independent experiments were carried out in triplicate. The MIC is defined as the lowest concentration of MD that fully inhibits the growth of the microorganism in the microdilution wells as detected by an unaided eye. The MBC is defined by registering the lowest concentration of MD that reduces the viability of the starting bacterial inoculum by ≥99.9% (CLSI guidelines). The results obtained were confirmed by using the Alamar Blue^®^ (AB) (Thermo Fisher Scientific, Waltham, MA, USA) planktonic susceptibility assay, as previously reported [38,39].

### 3.3. Determination of Minimal Biofilm Inhibitory Concentration (MBIC)

The antibiofilm activity of MD was measured by the MBIC assay in 96-well polystyrene microtiter plates. The wells were filled with MHB containing the broth cultures (10^5^ CFU/mL) and MD (0.5–1.5 mg/mL), and were incubated at 37 °C for 24 h. After the incubation, the supernatants were removed and the biofilms were rinsed in phosphate-buffered saline (PBS), and AB was diluted in sterile 0.01 M phosphate-buffered saline (PBS NaCl 0.138 M, KCl 0.0027 M; pH 7.4; Sigma-Aldrich, Milan, Italy) at 10% and added to the biofilms following the manufacturer’s instructions. The plates were incubated for 1 to 4 h at 37 °C; then, the percent reduction of AB was determined. Therefore, the colony-forming unit counting method was carried out to evaluate bacterial cell viability in the biofilm. Then, 100 µL of samples, taken from the MBIC wells, were used for CFU counts. Serial dilutions of the stock were performed in phosphate-buffered saline (PBS) (pH 7.2) and plated on MHA. The plates were incubated at 37 °C for 18–24 h. The same evaluation was performed in the case of the AB assay because of its non-toxic nature.

### 3.4. Coating of the Sutures with the MD

In this study, three kinds of sterile surgical sutures were used to produce an antimicrobial suture by coating: one non-absorbable multifilament Ethicon^®^Perma-hand silk suture composed of an organic protein (fibroin) with 0.35 mm of diameter, and two internal absorbable multifilament sutures: a Safil^®^ Quick synthetic braided and coated suture made of 100% polyglycolic acid (PGA) with 0.2 mm of diameter, and Ethicon^®^-coated vicrylrapide synthetic suture composed of a copolymer of 90% glycolide and 10% L-lactide with 0.15 mm of diameter. Following a previously reported experimental procedure [29,36,40], the sutures were cut under sterile conditions to obtain filaments that were 1.5 cm in length. Then, the sutures were dipped into the MD, followed by a drying period of 2 h at 37 °C. The weight of each coating on the multifilament sutures was measured by means of an analytical balance (XP205 Analytical Balance, Mettler Toledo, Columbus, OH, USA), before and after dipping/drying, and the resulting drug concentration was expressed per unit of length after this procedure. The amount of MD on each suture ranged from 2 to 7 mg/cm and thus was comparable or higher after dilution into the broth cultures to the MBIC values registered for *S. aureus* and *E. faecalis* (Table 1).

### 3.5. Antimicrobial and Antibiofilm Activity of the MD-Treated Sutures

The antimicrobial activity of sutures coated with MD was determined in MHA through the agar diffusion test. According to CLSI criteria, suspensions of 1.0 × 10^5^ CFU/mL of *S. aureus* and *E. faecalis* were prepared as mentioned above. Then, 200 μL of the suspension were plated uniformly on MHA plates and MD-treated, and untreated sutures were placed onto the surface. Then, the plates were incubated at 37 °C for 24 h.

The antibiofilm activity of sutures coated with MD was tested through the bacterial adhesion assay. Broth cultures of *S. aureus* and *E. faecalis* at the concentration of 1.0 × 10^5^ CFU/mL were prepared as mentioned above. The MD-treated and untreated sutures were inoculated with 2 mL of broth cultures dispensed in six-well polystyrene microtiter plates (Eppendorf, Hamburg, Germany). Then, the plates were incubated at 37 °C for 24 h in static conditions.

### 3.6. Scanning Electron Microscopy (SEM)

Scanning electron microscopy (SEM) was employed to analyze bacterial adhesion and biofilm formation on MD-coated and untreated sutures. The sutures were rinsed with 0.1 M of phosphate-buffered solution (PBS) at pH 7.1 and fixed overnight with a 4% PBS–paraformaldehyde solution at 4 °C. Samples were further washed with PBS buffer and dehydrated using an ascending alcohol series before mounting onto aluminum stubs and gold sputtering in an Emitech K 550 (Emitech Ltd., Ashford, Kent, UK).

The samples were imaged by means of SEM with LaB6 electron beam (Zeiss EVO 50 XVP, Carl Zeiss SMY Ltd., Cambridge, UK), coupled to tetra solid-state BSE detector. SEM operating conditions comprehended 10 kV of accelerating voltage, a 10-mm working distance and a 1.2-nA probe current. The observations were performed under variable pressure at 0.75 torr using both the BSE and SE1 detectors. The images were captured with a line average technique using 20 scans.

### 3.7. Confocal Laser Scanning Microscopy (CLSM) Analysis for Visualization of Adherent Bacteria

Live/dead staining and confocal laser scanning microscopy (CLSM) investigated bacterial viability and biofilm formation on MD-treated and untreated sutures. The surgical suture samples were washed in PBS to selectively remove the non-adhered bacteria, and stained with BacLight kit (Thermo Fisher Scientific, Waltham, MA, USA) for 15 min at room temperature [41]. Then, sutures were washed in PBS and visualized using a Zeiss LSM510 META confocal system (Carl Zeiss, Jena, Germany) coupled to an inverted Zeiss Axiovert 200 microscope equipped with a Plan Neofluar oil-immersion objective (100×/1.45 NA). The green and red emission (SYTO 9 and propidium iodide, respectively) were excited using the 488 nm (4% of potency) of an argon laser and a helium/neon 543 nm source (2.5% of potency). To separate the fluorescence emissions, HTF 488/543 and NTF 545 as primary and secondary dichroic mirrors were used. Detector band-pass filters were set over 505–530 and 565–615 ranges for the green and red emissions, respectively. To avoid signal bleed-through, images were alternatively acquired in multitrack mode using ZEN 2012 (Carl Zeiss).

### 3.8. Statistical Analysis

The statistical significance of difference between controls and experimental groups for the antimicrobial activity was evaluated using Student’s *t*-test. Statistical analysis was performed using GraphPad Prism™ (Version 5.00) software (GraphPad Software, San Diego, CA, USA). A *p*-value < 0.05 was considered as statistically significant.

## 4. Conclusions

The data obtained in the present study demonstrated that the MD is effective toward Gram-positive microorganisms such as *S. aureus* and *E. faecalis*. Moreover, it is also capable of inhibiting biofilm formation, even when applied to suture threads. Therefore, surgical sutures coated with the MD have the potential to reduce SSIs as well as the risk of biofilm formation post-surgery, avoiding the use of additional antibiotic therapies with consequent weakening of the patient.

## Figures and Tables

**Table 1:** Evaluation of the minimum inhibitory concentration (MIC), minimum bactericidal concentration (MBC), and minimum biofilm inhibitory concentration (MBIC) of the medical device (MD) against *S. aureus*, *E. coli*, and *E. faecalis.*

Bacterial Strains	MIC (mg/mL)	MBC (mg/mL)	MBIC (mg/mL)
*S. aureus* ATCC 29213	2	4	1.5
*E. coli* ATCC 25922	>8	>8	n.t.
*E. faecalis* ATCC 29212	2	4	1

n.t.: not tested.
